# New Appointments

**Published:** 1852-12

**Authors:** 


*New Appointments.* — Dr. J. Lawrence Smith, Prof. of Chemistry in the College of Louisiana, has been chosen Professor of Chemistry and Materia Medica, in the University of Virginia, in the place of Dr. Robert E. Rogers, recently called to the same chair in the University of Pennsylvania.

The regents of the University of Michigan, in July last, elected Dr. A. B. Palmer, of Chicago, Illinois, Professor of General, Descriptive, Morbid and Microscopic Anatomy, in the Medical Department.

In the Philadelphia Medical College several changes have been made. Prof. Thomas Spencer, formerly connected with Geneva Med. College, has been appointed to the chair of Materia Medica and Pathology. Dr. Henry

Geiger takes the chair of Midwifery and Diseases of Women and Children. Dr. Vandyke has been transferred to the chair of Practice, in the place of Prof. Thomas D. Mitchell, lately elected to the corresponding chair in the Kentucky School of Medicine. Dr. Bryen has been transferred from the chair of the Institutes of Medicine, to Surgery.

Dr. John A. Swett, has been appointed Professor of Clinical Medicine in the New York College of Physicians and Surgeons.

Dr. L. M. Lawson, of the Medical College of Ohio, has been transferred to the chair of the Theory and Practice of Medicine, made vacant by the recent death of Prof. Drake. Prof. Lawson previously held the chair of Physiology and Pathology.

